# Toward a Natural History of Team Sports

**DOI:** 10.1007/s12110-018-9322-6

**Published:** 2018-06-16

**Authors:** Kevin M. Kniffin, Michelle Scalise Sugiyama

**Affiliations:** 1 S. C. Johnson College of Business, Dyson School of Applied Economics and Management, Cornell University, Ithaca, NY 14853 USA; 2 University of Oregon, Eugene, OR USA

**Keywords:** Team sports, Team play, Play fighting, eSports

## Why Play in Teams?

Play and play fighting are human universals (Brown 1991:140), and certain types of play appear to recur across human cultures regardless of social or technological complexity (Cooper 1949; Culin 1907; Murdock 1945). Despite this, little attention has been paid to the question of whether these behaviors are adaptations or by-products. Do these behaviors develop spontaneously, or are they learned? Do they exhibit evidence of design and function, or do they emerge from the operation of physical and psychological structures that evolved for other purposes? The articles in this special issue of *Human Nature* are dedicated to piquing curiosity about and stimulating research on these questions.

One clue to the nature of play is its ubiquity: play (including play fighting) occurs across a broad range of taxa, suggesting that it is a deep part of our zoological heritage. Thus, one potentially fruitful line of inquiry is cross-species comparison, which can be used to tease out those aspects of play behavior that are unique to humans and those that we share with other species. On this point, it has been noted that play tends to occur in endothermic vertebrate species that exhibit postnatal parental care (Bekoff and Byers 1998; Ficken 1977) and tends to occur largely—although by no means exclusively (Cordoni 2009; Logan and Longino 2013; Pellis and Iwaniuk 1999)—during the juvenile period (Fagen 1981). These patterns suggest a relationship between play and ontogeny and inform the view in ethology that play is an adaptation that develops, rehearses, and/or fine-tunes skills that provide fitness benefits later in the individual’s lifespan (Fagen 1974, 1981). Others have noted that play provides opportunities for comparative self-assessment, enabling individuals to track their progress toward mastery of a given skill and their proficiency relative to that of their fellows (e.g., Thompson 1998). Onlookers, too, may assess the absolute and relative proficiency of players (e.g., Apostolou 2015; Apostolou and Zacharia 2015). The evaluative opportunities presented by play may explain its persistence into adulthood, especially among long-lived species that form cooperative alliances and/or exhibit dominance hierarchies.

One apparently unique feature of human play is play in teams. In nonhuman species, play behavior is overwhelmingly dyadic. Even in gregarious taxa, group play appears to be rare. When it does occur (e.g., in bonobos; Behncke 2016), it tends to be polyadic rather than coalitional: although several individuals may participate, their interactions are one-on-one rather than group-on-group. With the possible exception of dolphins (Scalise Sugiyama et al. 2018), humans are the only species known to play in teams (Symons 1978:191). Moreover, there are hints that this behavior is species-typical: research indicates that team sports are pervasive across forager (Scalise Sugiyama et al. 2018) and industrialized (Deaner and Smith 2013) societies, suggesting that team play is scaffolded by powerful motivational architecture.

Humans also appear to be the only species that play-fights in teams. Although definitions of combative play vary somewhat (Deaner and Smith 2013; Sipes 1973), the similarities between sports and fighting have long been noted (e.g., Fenichel and Rangell 1995; Lorenz 1966; Murdock 1949; Tiger 1970). As with play in general, dyadic play fighting is common across a wide range of species (Boulton and Smith 1992), but team play fighting is not, raising the question of why this particular form of team play is so widespread across human groups. Another striking feature of combative team play is the powerful emotional responses it evokes in non-participants. Human interest in team contact sports goes well beyond a desire to participate: humans also exhibit (i) a keen interest in watching teams play (spectatorship), (ii) a predilection for evaluating (e.g., sports statistics, fantasy football, schoolyard picking; see Barbaro et al. 2018) and criticizing (e.g., trash-talking; Kniffin and Palacio 2018) the comparative skills of players, (iii) strong and public preferences (e.g., loyalties; Kruger et al. 2018) for certain teams over others, and (iv) pronounced emotional and physiological responses to perceived officiating bias (e.g., Alvarado et al. 2018).

Whence the motivation for these behaviors? This question is a challenging one because combative team play is a hybrid that combines motor play, strategic play, and social play (i.e., play in which individuals interact with each other). Combative team play requires that individuals coordinate their actions with their team members in ways that are calculated to outmaneuver the opposing team, which in turn requires anticipating the actions of opponents. This complex activity may thus engage psychological structures associated with cooperation, planning, theory of mind, and in-group/out-group bias, among others. These components of combative team play are a logical starting point for questions regarding design and function. For example, are the cooperative challenges involved in team play fighting specific to this activity or are they typical of cooperation in general? Is the strategic planning deployed in combative team play applicable only in this context, or is it generalizable to other spheres of human endeavor? As these questions suggest, the study of combative team play may shed new light not only on play behavior but on the psychological phenomena involved in its production.

We believe that team contact play merits more attention from evolutionary social scientists and biologists. The present volume directs attention to some of the distinctive aspects of this behavior and encourages future research aimed at a more nuanced and holistic understanding of this zoologically unusual phenomenon. For example, the common use of language such as “combative sports” (Sipes 1973:68) and “combat” sports (Deaner and Smith 2013:8) in the play literature implies recognition of an affinity between contact sports and warfare. This raises questions about the nature of this relationship—i.e., the behavioral and contextual cues evoked by team contact play that invite a comparison with combat, explored herein by Scalise Sugiyama et al. (2018). Parallels between contact sports and combat may also be relevant to understanding other findings reported in this issue, such as the hormonal profiles associated with team sports participation and spectatorship (Alvarado et al. 2018; McHale et al. 2018), preferences for more-proficient players as coalition members in combat (Barbaro et al. 2018), and increased self-perceived mate value following victory in a sporting competition (Longman et al. 2018). Another parallel is suggested by the observation that value systems related to war and sports “tend to overlap and support each other’s presence” (Sipes 1973:65). This line of inquiry is developed in Pollack et al.’s (2018) study of belief in supernatural assistance and its influence on combat outcomes, as simulated through team contact play. Other contributions explore non-physical avenues and expressions of competition in sports, such as the use of “trash talk” to denigrate opponents (Kniffin and Palacio 2018) and the related phenomena of team rivalry and fan loyalty (Kruger et al. 2018). Although (with the exception of Scalise Sugiyama et al. 2018) the studies in this issue focus on sports played in modern nation-states, their findings invite us to imagine the challenges and opportunities presented by ancestral human environments that have resulted in the development and persistence of team contact play across human societies.

## Sports Teams as Organizational Laboratories

This issue also calls attention to the value of using sports as a model domain for studying evolutionary dynamics within and among teams. The contributions herein point to a range of theoretical questions that can be profitably explored and tested through the use of sports teams and related phenomena (e.g., spectatorship, intergroup rivalries, virtual sports), as well as methodological innovations that are increasingly showcased by the study of sports. The high number, diversity, and accessibility of sports teams—as organizations unto themselves—offers a tremendous opportunity for researchers interested in exploring how organizations work.

To date, sexual selection theory has been central to research on motivational factors underlying sports participation and spectatorship. For example, using a sample of male professional tennis players, Farrelly and Nettle (2007) test the prediction that if men are ultimately motivated (Buss 2015) to succeed in sports for the benefit of reproductive success, then marriage will tend to be associated with a reduction in performance. Other research informed by this theoretical perspective suggests that the substantially higher variance in reproductive fitness available to men compared with women helps account for apparently higher levels of male competitiveness, often manifested in the domain of team sports (e.g., Apostolou and Lambrianou 2017; Balish et al. 2016; Deaner et al. 2012, 2016). Recent work replicates the overall between-sex difference in between-sex competition but otherwise finds that women do tend to be as competitive as men when competing against themselves (Apicella et al. 2017). The increasing trend toward coed sports competitions (e.g., curling in the 2018 Winter Olympics had a new mixed-gender competition [Costa 2018]) adds further dimensions to explore using this theoretical framework.

Team sports also provide an arena for testing multilevel selection theory. For example, Kniffin and Wilson (2005, 2010) showcase this theory in relation to a field study of rowing teams. Owing to the nature and rules of the sport, rowers exhibit phenotypic uniformity with each other; consequently, they meet one of the primary criteria needed for variation-and-selective-retention to occur at the organizational level of teams (as specified by Wilson and Sober 1994). Dawkins (1976) examined rowers in support of his reductionist approach wherein team-level performance is universally recognized as epiphenomenal artifacts of individual pursuits. Although the levels-of-selection debate is prone to seemingly endless definitional arguments (e.g., Wilson and Kniffin 2003), team sports offer concrete opportunities for theory development and hypothesis testing in relation to understanding the ways in which individuals interact with groups.

For application of both sexual and multilevel selection theoretical frameworks, team sports provide dynamic “organizational laboratories” replete with increasingly accessible and progressively finer-grained data (complementing Kahn’s 2000 recognition of the sports industry as a “labor market laboratory”). For example, taking advantage of salary information that tends to be publicly available for high-profile sports (and for which performance data is also available), numerous researchers have examined the degree to which conflicts exist over how teams compensate members in relation to each other (e.g., Bloom 1999; Kniffin 2009; Trevor et al. 2012). At the individual level, Frank (2012) has found that people tend to be willing to trade relatively higher status in exchange for relatively lower pay (and vice versa) when working as part of a close-knit team. In light of this finding, it is sensible that current research is examining the conditions under which (and the degree to which) teams can benefit as a function of how individual members are compensated (in money or other important currencies; Kniffin 2006) in relation to each other. Fewell et al. (2012), for example, report that NBA teams with more phenotypic uniformity in the distribution of ball-touches (an important currency among players) tended to outperform teams whose plays tended to rely disproportionately on a smaller number of higher-profile players. Given the wide variety of rules and incentive structures across sports (Katz 2001), the lack of consensus with respect to whether and how team-level allocation of resources might affect team-level performance is not surprising. However, the fact remains that—as long as one recognizes sport-specific rules and incentive structures—team sports offer public “test sites” for examining the relative importance of individual- and team-level interests.

The public nature of contemporary team sports also provides an arena for considering the influence of “nature” and “nurture” in successful competitive behavior. Studies regarding the social dynamics of sports typically treat biological variation as nonexistent. For example, in a study comparing first-ballot Hall of Fame baseball players with other Hall of Fame players, Cotton et al. (2011) find that players elected on the first ballot (i.e., elite among elite) tend to have richer developmental mentorship networks than the comparison group. Although this finding is sensible and insightful, no consideration is given to the possibility that biological differences exist between the two samples. Of course, the application of biological perspectives to the study and management of team sports has been muddled by now-discredited perspectives on race (Hoberman 1997), but contemporary work on the subject of gene-environment interactions is markedly more sophisticated for questions focused on sports (e.g., Epstein 2014) as well as broader scopes (e.g., Freese 2008; Freese and Shostak 2009). In this special issue, three of the contributions (Alvarado et al. 2018; Longman et al. 2018; McHale et al. 2018) examine biological relationships between hormones and team sports participation and spectatorship, generating insights that would obviously have been impossible if one were to assume that biological variation is either nonexistent or irrelevant.

Team sports are attractive subjects of study because of their relative accessibility to researchers and their naturalistic settings, but it is also true that the examination of sensitive or carefully controlled questions benefits from the employment of field-based simulations or laboratory experiments. Here, Pollack et al. (2018) and Kruger et al. (2018) present findings from field-based simulations, and Barbaro et al. (2018) report findings from a lab experiment. In all three cases, the research design entailed realistic scenarios where slight but controlled variations allowed for between-subjects comparisons to conditions where participants were randomly assigned. Pollack et al.’s test has relevance for any sports fan who has watched elite players credit the supernatural for their success. Similarly, Kruger et al. offer new perspectives on the dynamics that result when sports fans wear apparel bearing the symbolically laden colors and logos of their favorite team.

The use of team sports as a model domain for studies of behavior and psychology also provides an opportunity to demonstrate the value of evolutionary thinking for contemporary commercial or applied purposes (e.g., Roberts et al. 2012). Kruger et al.’s study of the ways in which team-based apparel can influence the real-world interactions of its wearers has both profit and safety implications. For example, although Kruger et al. do not examine instances of rage or anger among fans, their findings offer valuable baseline evidence for researchers and policymakers interested in better understanding and preventing the kind of lethal violence that can occur when fans of a given team are identified at a rival team’s home arena (e.g., Swenson 2012).

Barbaro et al.’s findings, which examine the impact of video-game outcomes on interpersonal decision-making, complement the massive and rapid growth of “eSports.” This development includes the growth of platforms, such as Twitch, that draw millions of people to watch others play in various competitions. It also includes the emergence of team-based virtual competitions: for example, the National Basketball Association has commissioned its own “mirror” league of teams that play a popular basketball video game (Funk et al. 2018). As indicated by Kasumovic et al.’s (2015) application of evolutionary perspectives to the perennial question of why people play violent video games, this type of play is ripe for further investigation. The new frontier of virtual-team game play offers the opportunity for substantial scaling and provides an arena for exploring issues of interest to both academics and game developers (e.g., increasing inclusivity across gender, class, and ethnic barriers; see Mikulak 2015).

Two buzz-phrases commonly associated with the study of sports are “sports analytics” that utilize “big data.” Typical topics for analytics include player evaluation (e.g., Kniffin et al. 2017; Lewis 2004), team-level strategy assessments (Alamar 2013), and challenges to various concepts such as “momentum” (e.g., Kniffin and Mihalek 2014; Tversky and Gilovich 1989). Both Kruger et al.’s and Barbaro et al.’s work can serve as baselines for scaled-up research, and Kniffin and Palacio’s study of trash talk lends itself to “big data” collection, either through systematic collection and analysis of social media postings (e.g., Margolin and Liao 2018) or through new technologies that would allow unrestricted collection of audio communication among players. Kovalchik and Reid’s (2018) study of tennis players is instructive: their use of “machine learning” methods, whereby players’ emotions can be successfully classified on a play-by-play basis through computer detection, highlights the unanticipated ways in which new technology can benefit researchers as well as players and coaches. In sum, in alignment with our overall goal to cultivate more consideration and analysis of sports from multidisciplinary perspectives informed by evolutionary thinking, this special issue showcases numerous windows for further explorations via the study of team sports that employ a wide and growing array of methodological and analytical tools.
